# Virulence-related traits of epidemic *Acinetobacter baumannii* strains belonging to the international clonal lineages I-III and to the emerging genotypes ST25 and ST78

**DOI:** 10.1186/1471-2334-13-282

**Published:** 2013-06-20

**Authors:** Maria Giannouli, Luísa CS Antunes, Veronica Marchetti, Maria Triassi, Paolo Visca, Raffaele Zarrilli

**Affiliations:** 1 Department of Public Health, University of Naples “Federico II”, Naples, Italy; 2 Department of Biology, University “Roma Tre”, Rome, Italy

**Keywords:** Epidemiological typing, Biofilm formation, Resistance to desiccation, Cell adherence/invasion, *Galleria mellonella* infection

## Abstract

**Background:**

*Acinetobacter baumannii* is responsible for large epidemics in hospitals, where it can persist for long time on abiotic surfaces. This study investigated some virulence-related traits of epidemic *A. baumannii* strains assigned to distinct MLST genotypes, including those corresponding to the international clones I-III as well as emerging genotypes responsible for recent epidemics.

**Methods:**

Genotyping of bacteria was performed by PFGE analysis and MLST according to the Pasteur’s scheme. Biofilm formation on polystyrene plates was assessed by crystal violet staining; resistance to desiccation was evaluated on glass cover-slips when kept at room-temperature and 31% relative humidity; adherence to and invasion of A549 human alveolar epithelial cells were determined by the analysis of viable bacteria associated with or internalized by A549 human alveolar epithelial cells; *Galleria mellonella* killing assays were used to analyze the virulence of *A. baumannii in vivo*.

**Results:**

The ability to form biofilm was significantly higher for *A. baumannnii* strains assigned to ST2 (international clone II), ST25 and ST78 compared to other STs. All *A. baumannii* strains survived on dry surfaces for over 16 days, and strains assigned to ST1 (international clone I) and ST78 survived for up to 89 and 96 days, respectively. Adherence to A549 pneumocytes was higher for strains assigned to ST2, ST25 and ST78 than other genotypes; a positive correlation exists between adherence and biofilm formation. Strains assigned to ST78 also showed significantly higher ability to invade A549 cells. No significant differences in the killing of *G. mellonella* worms were found among strains.

**Conclusions:**

Elevated resistance to desiccation, high biofilm-forming capacity on abiotic surfaces and adherence to A549 cells might have favoured the spread and persistence in the hospital environment of *A. baumannii* strains assigned to the international clones I and II and to the emerging genotypes ST25 and ST78.

## Background

*Acinetobacter baumannii* is an emerging bacterial pathogen responsible for widespread and persistent outbreaks among hospitalized patients. *A. baumannii* infections are difficult to control since many epidemic strains can resist several classes of antibiotics and disinfectants and are able to contaminate abiotic surfaces of the hospital environment, including the medical equipment [1,2]. The vast majority of *A. baumannii* epidemics are caused by a limited number of strains worldwide, belonging to the initially named European clones, but now regarded to as International clonal lineages (IC) I, II and III [3-5]. Additional *A. baumannii* genotypes have recently emerged as epidemic clones in some regions, such as the closely related ST15 and ST84 genotypes from Europe [3,4], the ST25 genotype from Eurasia [4,6] and genotype ST78 isolated in several Italian hospitals [4,7]. Since the majority of epidemic strains are multi-drug-resistant (MDR) or extensively-drug-resistant (XDR) [8], many studies have investigated the genetic and functional basis of antimicrobial resistance [1,2,4-6]. In contrast, few studies are available on the virulence traits and pathogenic potential of *A. baumannii*. It has been reported that *A. baumannii* can form biofilms on several abiotic surfaces, including polystyrene, polypropylene, polytetrafluoroethylene and glass [9], and ca. 70% of strains investigated in multicentre prospective cohort study performed in 28 Spanish hospitals were biofilm-formers [10]. While no difference in biofilm formation was observed between outbreak and sporadic strains, IC-II strains seem to form larger biofilms than IC-I strains [11]. Biofilm formation appeared to be positively correlated with multidrug resistance [10], as well as with the expression of several virulence factors, including the outer membrane protein OmpA, the extracellular polysaccharide poly-β-(1,6)-N-acetyl glucosamine (PNAG), type I pili, a homologue of the staphylococcal biofilm-associated protein (Bap), the outer membrane protein CarO, a quorum sensing system and proteins involved in histidine metabolism, such as urocanase [9,12-17]. The ability of *A. baumannii* strains to survive for a long time on dry surfaces is likely to contribute to their persistence in hospitals [18]. In this respect, it has been recently reported that RecA protein is involved in general stress response and resistance to heat shock and desiccation in *A. baumannii*[19]. In addition, the ability of *A. baumannii* to adhere and invade epithelial cells has been investigated [20]. Among the virulence factors that may contribute to these processes, the Bap protein has been demonstrated to play a role during adherence to epithelial cells [21], and OmpA and phospholipase D have been shown to contribute to invasion of epithelial cells [15,22].

The majority of the above studies, however, have been performed using a limited number of strains, often ATCC 19606^T^ and/or ATCC 17978 reference strains [12,15,17,23]. Only a few studies have compared the virulence traits of ICs I-III [11,23,24], while no studies have analyzed the virulence features of other emerging epidemic lineages. Although comparative genome analysis of twelve *A. baumannii* strains assigned to distinct genotypes [25,26] and of 136 *A. baumannii* strains belonging to *A. calcoaceticus-baumannii* complex [27] has demonstrated that the genes encoding putative virulence factors are mostly conserved, phenotypic data on the expression of putative virulence factors disclosed variability among strains, even those belonging to the same IC, likely reflecting differences in expression levels [11,23,24].

The objective of the present study was to identify virulence-related traits of epidemic strains assigned to distinct genotypes of *A. baumannii* that could contribute to their ability to colonize and infect the human host and persist in the hospital environment. Biofilm formation, resistance to desiccation, adherence to and invasion of A549 human bronchial cells, and killing of *Galleria mellonella* caterpillars were evaluated for a diverse collection of MDR or XDR *A. baumannii* strains isolated during outbreaks in European hospitals, and in the reference strains ATCC 19606^T^ and ATCC 17978.

## Methods

### Bacterial strains, cell line and culture conditions

Twenty-three *A. baumannii* strains isolated during outbreaks that occurred in several Mediterranean hospitals [4,28-31], plus *A. baumannii* ATCC19606^T^ and ATCC17978 reference strains [32,33] were included in the study (Table 1). Epidemiological features of strains and number of patients involved in the outbreaks were in accordance to previous publications [4,28-31]. No ethical approval was required for the study because there was no access to patient data. Bacteria were routinely cultured in Luria-Bertani broth (LB). Genotyping was performed by pulsed-field gel electrophoresis (PFGE) and multilocus sequencing typing (MLST) analyses as previously described [3,31]. Antimicrobial susceptibilities were determined by a reference microdilution method [34]. MDR and XDR phenotypes were designed according to Magiorakos et al. [8]. The M9 minimal broth supplemented with magnesium sulphate and glucose as carbon source (M9) and Mueller Hinton broth (Oxoid, Milan, Italy) were used for biofilm growth [35] and pellicle formation [36], respectively. A549 human alveolar epithelial cells were cultured as previously described [15].

**Table 1:** **Epidemiological, phenotypic and genotypic data of the *A. baumannii *isolates included in the study**

**Strain**	**Isolate source**	**Hospital**	**Year**	**Patients**^**a**^	**PFGE type**	**MLST**	**MDR/XDR**	**IMP MIC mg/L**	**Reference**
AYE	Urine	Kremlin-Bicetre/FR	2001	12	A	ST1 (IC-I)	MDR	2	[28]
700	Blood culture	F-Naples/IT	1999	81	B	ST1 (IC-I)	MDR	2	[4]
2979	Wound swab	Agrigento/IT	2002	14	C	ST20 (IC-I)	MDR	2	[4]
3130	Blood culture	SG-Beirut /LB	2004	17	D	ST20 (IC-I)	XDR	16	[4]
2105	Bronchial aspirate	F-Naples /IT	2002	43	E	ST2 (IC-II)	XDR	16	[4]
3990	Central venous catheter	M-Naples /IT	2006	12	E	ST2 (IC-II)	XDR	32	[31]
ACICU	Cerebrospinal fluid	Rome/IT	2005	14	E1	ST2 (IC-II)	XDR	32	[30]
2735	Bronchial aspirate	M-Naples /IT	2004	2	E2	ST2 (IC-II)	XDR	64	[4]
3889	Bronchial aspirate	Athens/GR	2005	4	F	ST2 (IC-II)	XDR	16	[4]
4026	Bronchial aspirate	SJ-Beirut /LB	2007	5	G	ST2 (IC-II)	XDR	16	[4]
4009	Blood culture	Genoa/IT	2007	4	H	ST2 (IC-II)	XDR	32	[4]
4025	Bronchial aspirate	SG-Beirut /LB	2005	3	I	ST3 (IC-III)	XDR	16	[4]
LUH 5875	Blood culture	Utrecht/NL,	1997	15	J	ST3 (IC-III)	MDR	2	[29]
3868	Bronchial aspirate	Izmir/TK	2003	2	K	ST15	XDR	16	[4]
3871	Bronchial aspirate	Istanbul/TK	2003	1	K1	ST84	XDR	16	[4]
3890	Bronchial aspirate	Thessaloniki/GR	2003	12	L	ST25	XDR	16	[4]
3865	Blood culture	Kocaeli/TK	2005	47	M	ST25	XDR	64	[4]
4190	Blood culture	M-Naples /IT	2009	3	N	ST25	XDR	64	[4]
ATCC 19606^T^	Urine	Atlanta/USA	Before 1949	1	O	ST52	susceptible	2	[32]
ATCC 17978	Blood culture	France	1951	1	P	ST77	susceptible	2	[33]
3909	Bronchial aspirate	M-Naples /IT	2007	55	Q	ST78	XDR	16	[31]
3957	Bronchial aspirate	M-Naples /IT	2007	55	Q	ST78	XDR	16	[31]
3911	Blood culture	M-Naples /IT	2007	1	Q1	ST78	XDR	16	[31]

Abbreviations: F-Naples, Federico II University Hospital, Naples; M-Naples, Monaldi Hospital, Naples; SG-Beirut, Saint Gorge Hospital, Beirut; SJ-Beirut, Saint Joseph Hospital, Beirut; *FR*, France; *GR*, Greece; *IT*, Italy; *LB*, Lebanon; *TK*, Turkey; ^a^ Number of patients from whom an isolate with each particular PFGE type was detected; *MLST*, Multi-locus sequencing typing; *ST*, sequence type; *IC*, international clone; *IMP*, imipenem; *MIC*, Minimum inhibitory concentration.

### Measurement of biofilm formation

The ability of *A. baumannii* strains to form biofilm was measured using a microtiter plate assay [37]. One hundred microliter aliquots of overnight cultures were diluted to an optical density at 600 nm (OD_600_) of ~ 1.0. and dispensed in 96-well polystyrene microtiter plates. The number of bacterial cells in the supernatant was monitored by OD_600_ measurements during 24 h growth at 37°C. After removal of the culture, plates were washed with phosphate-buffer saline solution (PBS) and air-dried. Biofilm was stained with 0.1% crystal violet solution for 15 min. After washing with distilled water, the biofilm-associated dye was solubilised with 200 μl of 95% ethanol and the OD_540_ was measured. The OD_540_/OD_600_ ratio was used to normalize the amount of biofilm formed to the total cell content. In order to assess the induction of biofilm formation in the presence of antibiotics, the bacteria were grown for 24 h in microtiter plates in the presence of imipenem (Merck Research Laboratories, Rahway, N.J., USA) at sub-inhibitory concentrations as previously described [35]. Sub-inhibitory concentrations of imipenem were 0.5 mg/L for strains AYE, 700, 2979, LUH 5875, ATCC 19606^T^, and ATCC 17978, and of 4 mg/L for all other strains, which corresponded to 1/4 and 1/4 – 1/16 the MIC, respectively (Table 1). Three independent experiments, each one performed in triplicate, were conducted for each strain. For the pellicle formation at air-liquid interface assay, bacteria were grown for 72 h at 37°C without shaking, as previously described [36]. Positive air-liquid biofilm samples were identify visually; the isolates were considered positive when a pellicle was covering the whole liquid surface.

### Desiccation survival assay

The desiccation assay was performed as previously described [18]. One ml aliquots of overnight LB cultures were centrifuged at 11,600 × g for 5 min in a microcentrifuge. The cell pellet was washed twice with PBS and suspended in distilled water to an OD_600_ of 1.0. Twenty microliters of each suspension were deposited onto a glass cover slip to produce an inoculum of ~2 × 10^7^ colony-forming units (CFU). The coverslip was kept at 31% relative humidity by the presence of a saturated CaCl_2_ × 6H_2_O in an uncovered Petri dish, and stored at room temperature in an air tight transparent plastic box (17 × 11 × 5.5 cm) for up to 16 weeks. Strains were distributed into four separate boxes; each box stored up to six strains. Viable cell counts were determined once a week as previously described [23].

### Adherence to and invasion of A549 cells

Adherence of *A. baumannii* strains to A549 cells (human type 2 pneumocytes) was determined as described previously [15], with minor modifications. In brief, ~ 10^5^ A549 cells were infected with ~ 10^7^ bacterial CFU and incubated for 60 min at 37°C in 5% CO_2_ (v/v) atmosphere. Non-adherent bacterial cells were removed by washing with PBS. Infected cells were lysed by the addition of 1 ml distilled water and serial 10-fold dilutions were plated on LB agar to determine the number of CFU of adherent bacteria. To determine adherent and invading bacteria, A549 cells were infected with *A. baumannii* strains as described above. The monolayers were then treated with 1 ml of fresh culture medium containing 5 mg/L of colistin sulfate (Sigma-Aldrich, Milan, Italy) for further 30 min, the shortest time point that resulted in the killing of all extracellular bacteria added to the monolayers. All strains included in the study were killed after a 30 min treatment with 5 mg/L of colistin. Afterwards, the cells were washed with PBS, harvested with trypsin, and lysed with sterile distilled water. Dilutions from harvested samples were inoculated on LB agar plates and bacterial colony counts were estimated after overnight incubation at 37°C. Each experiment was performed in triplicate.

### *G. mellonella* killing assay

The virulence of *A. baumannii in vivo* was assessed using the *G. mellonella* insect model of infection [23], with minor modifications. Ten *G. mellonella* caterpillars (average weight 500 ± 60 mg) were injected with 10 μl of serial ten-fold dilutions in PBS of *A. baumannii* cells grown overnight at 37°C in LB. Bacterial colony counts (CFU/ml) on LB agar plates were used to estimate the number of viable cells in each inoculum. Ten larvae that received no injection and ten larvae injected with 10 μl PBS were used as negative controls of each experiment. Two independent experiments, each one performed in triplicate (thirty larvae for each strain), were conducted for each strain. Larvae were incubated at 37°C in Petri dishes (ten larvae per dish) and monitored every 12 h for a total of 72 h. Survival curves were plotted using the Kaplan-Meier estimator and expressed in percentage. Lethal dose 50% (LD_50_) values were calculated using GraphPad Prism v.5.04 (GraphPad Software, La Jolla California USA) according to the following equation: Y=  A + (1-A)/(1 + e^(B–G×lnX)^), where X is the number of viable bacterial cells injected, Y the fraction of larvae killed by a given bacterial inoculum, A is the fraction of larvae killed by the control solution, and B and G are curve-fitting constants automatically calculated by GraphPad Prism [23]. LD_50_ values were calculated as the value of X that corresponds to Y = 0.5.

### Statistical analysis

Data were analysed using Statistical Package for the Social Sciences Version 13.0 (SPSS Inc., Chicago, IL, USA). The significance of the induction of biofilm formation in the presence of imipenem was analyzed with the Student’s *t* test and induction was considered significant when the P value was <0.05. The variability of strains assigned to the same ST was investigated using the One-Way Analysis of Variance (ANOVA), in which strains were grouped according to ST and differences were considered significant when the P value was <0.05. Correlations were evaluated by regression analysis using the Pearson’s correlation coefficient (r).

## Results

### Biofilm formation

Twenty-three *A. baumannii* strains isolated during outbreaks that occurred in several hospitals in France, Greece, Italy, Lebanon, Netherlands and Turkey [4,28-31], and *A. baumannii* ATCC19606^T^ and ATCC17978 reference strains [32,33] were investigated. The strains had previously been assigned to 10 different MLST sequence types (STs) and to 17 different major PFGE types. The collection includes strains assigned to major international clones IC-I, IC-II and IC-III and additional emerging genotypes ST15, ST25 and ST78. The majority of the strains were representative of cross-transmission episodes and were isolated with identical PFGE type from more than two patients of the same or different institutions. Strains 3909 and 3957 assigned to ST78 were isolated with identical PFGE type Q from different patients during the same inter-hospital outbreak [31]. Seventeen strains exhibited an imipenem minimum inhibitory concentration (MIC) ≥16 mg/L and were considered to be carbapenem-resistant and XDR; four strains were classified as MDR (Table 1).

**Figure 1:**
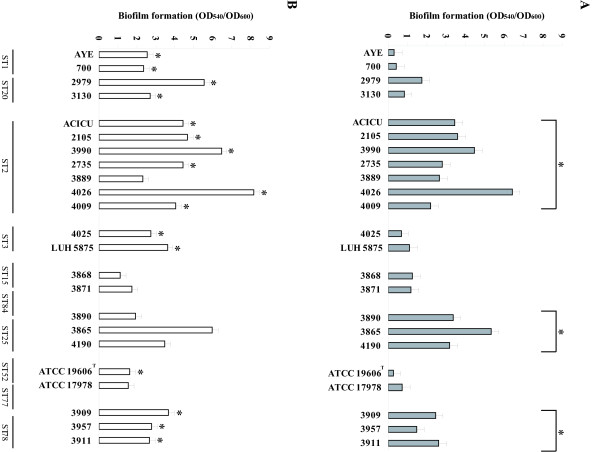
**Biofilm formation.** Biofilm formation on 96-well polystyrene microtiter plates by *A. baumannii* grown statically in M9 medium for 24 hours in the absence (panel **A**) or presence (panel **B**) of imipenem concentrations 0.5 mg/L (strains AYE, 700, LUH 5875, 2979, ATCC 19606^T^ and ATCC 17978), or 4 mg/L (all other strains). Strain and ST types are indicated on the bottom of the figure. Error bars represent standard deviations based on three independent experiments. Asterisks indicate statistically significant differences in biofilm formation among different STs (panel A) (p <0.05) and statistically significant induction in biofilm formation upon exposure to sub-MIC imipenem concentrations (panel **B**) (p <0.05).

The ability to form biofilm on polystyrene microtiter plates was evaluated for outbreak *A. baumannii* strains assigned to different PFGE genotypes and STs (Table 1). Strains assigned to the four major PFGE types E-H (ST2, corresponding to IC-II), PFGE types L-N (ST25), and PFGE type Q (ST78) formed significantly higher biofilm levels than strains assigned to other PFGE types (including ST1, ST20, ST3, ST15, ST84, ST52, and ST77) (p <0.05) (Figure 1A). No relevant differences in growth yelds were observed among the strains, thus excluding the possibility that difference in biofilm formation were due to difference in bacterial growth. In addition, *A. baumannii* strains assigned to ST2, ST25 and ST78 formed a robust biofilm pellicle at the air-liquid interface when grown at 37°C for 72 h without shaking (data not shown).

Since exposure of *A. baumannii* to sub-inhibitory concentrations of imipenem has been demonstrated to stimulate biofilm growth [35], a biofilm formation assay was performed in the presence of sub-inhibitory concentrations of the antibiotic (Figure 1B). Growth yields were not affected by imipenem at the used concentrations for all strains tested. Compared with the untreated control, significantly higher biofilm levels were observed after exposure to imipenem for strains assigned to ST2 (except strain 3889), and ST78, which already produced high biofilm levels before treatment, and for strains assigned to ST1 and its single locus variant ST20 (IC-I), to ST3 (IC-III) and to ST52 (ATCC 19606^T^) (p <0.05) which, conversely, were weak producers when grown in the absence of antibiotic. On the other hand, no significant differences in biofilm formation were observed for the strains assigned to ST15, ST84 ST25 and ST77 (Figure 1A and B).

### Resistance to desiccation

**Figure 2:**
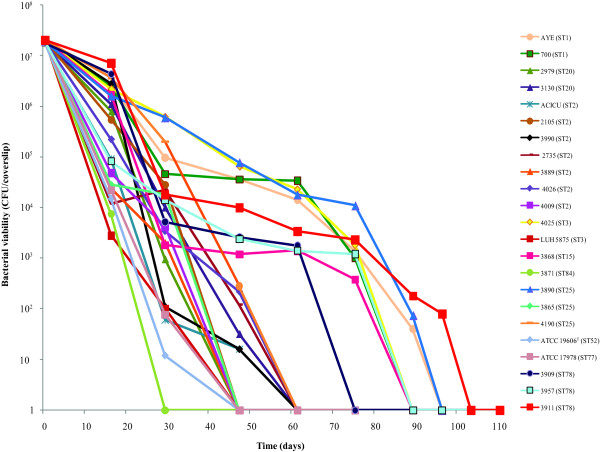
**Resistance to desiccation.** Resistance to desiccation of *A. baumannii* strains. Strains were inoculated onto rounded glass cover slips and incubated at 31% relative humidity and room temperature. A starting bacterial inoculum of ~ 2 × 10^7^ per cover slip was used. Values represent the mean of three independent experiments.

Strains showed an overall ability to resist desiccation, though survival times ranged from 16 to 96 days depending on the strain (Figure 2 and Additional file 1: Table S1). In particular, strains assigned to ST1 and ST78 showed extremely high resistance to desiccation, with survival times of 75 to 89 days and 68 to 96 days, respectively. Strains assigned to ST15, strain 4025 (ST3) and strain 3890 (ST25) showed a comparable trend of high resistance to desiccation, with survival times of 75 to 89 days. Strains assigned to ST2 and ST20, strains 4190 and 3865 (assigned to ST25) and strain ATCC 17978 were moderately resistant to desiccation with survival times of 33 to 54 days. Finally, strains 3871 (assigned to ST84), LUH 5875 (assigned to ST3) and ATCC 19606^T^ were less resistant to dry conditions, and survived for < 29 days (Figure 2 and Additional file 1: Table S1).

### Adherence to and invasion of A549 human bronchial cells

**Figure 3:**
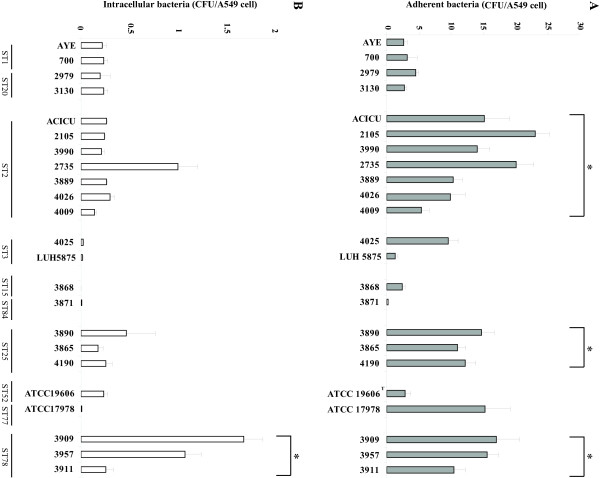
**Bacterial adherence to and invasion of A549 bronchial epithelial cells.** (**A**) Cell surface-associated bacteria after 60 min incubation at 37°C. Statistically significant differences (p <0.05) among different STs are indicated with asterisks. (**B**) Intracellular bacterial counts (CFU/well) obtained after 60 min incubation of A549 cells with bacterial strains and subsequent treatment for 30 min at 37°C with colistin (5 mg/L). Statistically significant differences among different STs (p <0.05) are indicated with asterisks. Each value represents the mean ± standard deviation (error bar) of three independent experiments performed in duplicate.

Different steps of the interaction between *A. baumannii* and A549 human alveolar epithelial cells were investigated, including adherence to epithelial cells and subsequent invasion [15]. *A. baumannii* strains displayed different ability to adhere to epithelial cells (Figure 3A). In particular, all strains assigned to ST2, ST25, ST78 and strain ATCC 17978 adhered to epithelial cells at significantly higher levels than other strains (p <0.05). Moreover, a highly significant correlation was found between adhesiveness of *A. baumannii* to A549 human alveolar epithelial cells and biofilm formation for all strains, except ATCC 19606^T^ and ATCC 17978 (r = 0.832, p <0.01).

Strains assigned to ST1, ST20, ST2, ST25 and ST78 and reference strain ATCC 19606^T^ were all invasive, although only strains assigned to ST78 showed significantly high levels of invasiveness when compared with other genotypes (p <0.05) (Figure 3B). A similar number of bacteria adhered to A549 cells when the monolayers were incubated with *A. baumannii* strains for 60 min at 4°C, i.e. under conditions that do not allow for tissue invasion (data not shown).

### *G. mellonella* killing assays

The LD_50_ values were calculated for each strain at 24, 48 and 72 h post-infection (Table 2). For all strains, the LD_50_ decreased with post-infection time, attaining the final value at 72 h (Additional file 2: Figure S1). No relevant difference in larval death was observed at the subsequent time points (data not shown). Differences in LD_50_ between 24 h and 72 h provide a trend of the killing rapidity for individual strains (Table 2 and Additional file 2: Figure S1). The LD_50_ at 72 h were comprised between 8.1 × 10^4^ and 4.7 × 10^6^ CFU/larva, with the average LD_50_ being 5.8(±9.5) × 10^5^ CFU/larva. These values are in the range of those determined in a previous study on clinical *A. baumannii* isolates [23]. Strain-specific differences between LD_50_ calculated at 72 h were significant (p <0.001). In particular, strains ATCC 19606^T^ (ST52) and 3868 (ST15) had significantly (p <0.001) higher LD_50_ than all the other strains tested. In contrast, ST-specific differences between LD_50_ calculated at 72 h were not significant (p ≥0.05).

**Table 2:** ***G. mellonella *killing by *A. baumannii *strains**

**Strain**	**ST type**	**LD**_**50**_^**a**^			**Ratio 24/72 h**
		**24 h**	**48 h**	**72 h**	
AYE	ST1	2.8 (±0.3) × 10^6^	5.4 (±0.3) × 10^5^	2.3 (±0.1) × 10^5^	12.2
700	ST1	7.2 (±4.2) × 10^5^	2.7 (±0.5) × 10^5^	2.3 (±0.4) × 10^5^	3.1
2979	ST20	1.0 (±0.2) × 10^6^	6.6 (±0.2) × 10^5^	4.2 (±0.6) × 10^5^	2.4
3130	ST20	7.3 (±0.3) × 10^5^	5.8 (±0.6) × 10^5^	5.8 (±0.6) × 10^5^	1.3
2105	ST2	9.2 (±0.6) × 10^5^	8.2 (±0.5) × 10^5^	7.4 (±0.7) × 10^5^	1.2
3990	ST2	6.6 (±0.7) × 10^5^	1.1 (±0.2) × 10^5^	8.1 (±0.5) × 10^4^	8.1
ACICU	ST2	1.3 (±0.4) × 10^6^	7.4 (±0.3) × 10^5^	6.4 (±0.9) × 10^5^	2.0
2735	ST2	2.2 (±0.3) × 10^5^	2.0 (±0.1) × 10^5^	1.3 (±0.7) × 10^5^	1.7
3889	ST2	4.2 (±0.4) × 10^5^	2.4 (±0.1) × 10^5^	1.8 (±0.2) × 10^5^	2.3
4026	ST2	4.5 (±0.6) × 10^5^	2.7 (±0.5) × 10^5^	2.7 (±0.5) × 10^5^	1.7
4009	ST2	3.7 (±0.2) × 10^5^	3.2 (±0.7) × 10^5^	3.0 (±0.7) × 10^5^	1.2
4025	ST3	9.4 (±0.2) × 10^5^	2.7 (±0.9) × 10^5^	2.2 (±0.8) × 10^5^	4.3
LUH 5875	ST3	3.8 (±1.2) × 10^5^	2.8 (±0.2) × 10^5^	2.3 (±0.1) × 10^5^	1.7
3868	ST15	2.3 (±0.5) × 10^6^	2.3 (±0.6) × 10^6^	1.6 (±0.1) × 10^6^	1.4
3871	ST84	5.8 (±0.3) × 10^5^	3.9 (±0.5) × 10^5^	3.5 (±0.3) × 10^5^	1.7
3890	ST25	6.6 (±0.8) × 10^5^	4.6 (±0.3) × 10^5^	2.4 (±0.4) × 10^5^	2.8
3865	ST25	1.6 (±0.4) × 10^6^	1.3 (±0.1) × 10^6^	3.0 (±0.3) × 10^5^	5.3
4190	ST25	3.1 (±0.5) × 10^6^	2.1 (±0.3) × 10^6^	5.0 (±0.9) × 10^5^	6.0
ATCC 19606^T^	ST52	9.1 (±3.4) × 10^6^	6.7 (±0.7) × 10^6^	4.7 (±0.2) × 10^6^	1.9
ATCC 17978	ST77	6.7 (±0.1) × 10^5^	4.5 (±0.7) × 10^5^	3.1 (±0.6) × 10^5^	2.2
3909	ST78	2.0 (±0.4) × 10^6^	6.4 (±0.7) × 10^5^	6.4 (±0.7) × 10^5^	3.1
3957	ST78	3.3 (±1.2) × 10^5^	2.8 (±0.4) × 10^5^	2.5 (±0.6) × 10^5^	1.3
3911	ST78	7.8 (±1.9) × 10^5^	5.6 (±0.6) × 10^5^	1.4 (±0.6) × 10^5^	5.6

^a^LD_50_ values are expressed as CFU/larva.

## Discussion

Antibiotic resistance and long-term persistence in the hospital environment are two factors that most likely contribute to the success of *A. baumannii* as an opportunistic pathogen [1,2,5]. However, it is possible that the emergence of some epidemic strains or clones over others is the result of an improved ability to colonize patients and cause disease. In attempt to address this issue, biofilm formation, resistance to desiccation, adherence to and invasion of human epithelial cells and the ability of *A. baumannii* to kill *G. mellonella* catterpilars were investigated as possible virulence-related phenotypes among *A. baumannii* isolates belonging to the main ICs and to recently emerging clones [10,15,18,23]. To this purpose, a collection of 21 *A. baumannii* strains assigned to the three main lineages circulating worldwide, i.e. IC-I, II and III, as well as to distinct genotypes recently identified as responsible for outbreaks, and two reference *A. baumannii* strains ATCC 19606^T^ and ATCC 17978 were selected (Table 1).

Results demonstrate that epidemic *A. baumannii* genotypes differ in the ability to form biofilm on abiotic surfaces. In particular, strains assigned to genotypes ST2, ST25 and ST78 produce biofilm more efficiently and are able to form a robust biofilm pellicle at the air-liquid interface of the culture medium. In keeping with this result, it has been shown that strains belonging to the *A. baumannii-calcoaceticus* complex form higher biofilm pellicle than other *Acinetobacter* species [36] and that *A. baumannii* strains assigned to the IC-II form more biofilm than *A. baumannii* strains assigned to the IC-I [11]. It is also worth mentioning that the *A. baumannii* strain SMAL, responsible for two outbreaks in Northern Italy and shown to be an efficient biofilm producer [35], has recently been assigned to ST78 [7]. Biofilm could therefore be regarded as an important virulence feature for *A. baumannii* strains belonging to ST2, ST78 and also ST25. In consistence with previous findings [35], our study also demonstrates that exposure to sub-inhibitory concentrations of imipenem has an overall stimulating effect on biofilm formation as shown for epidemic strains assigned to ST1, ST2, ST3, ST20 and ST78. It is plausible that transition from planktonic to biofilm lifestyle upon imipenem exposure represents a defensive response of *A. baumannii* to the threat posed by the antibiotic challenge.

Most of the *A. baumannii* outbreak strains analyzed herein showed an impressive ability to survive on dry surfaces for long times, consistent with the overall capacity of this species to resist to drought [18,23,38]. Strain-dependent variation was found also for this character, since strains assigned to ST1 and ST78, as well as one strain assigned to ST25, showed extremely high resistance to desiccation; strains assigned to ST20, which belong to the same clonal complex as ST1, all ST2 strains, and two of three strains assigned to ST25 were moderately resistant to desiccation; in contrast, six other strains, including ATCC 19606^T^, were less resistant to drought. It has recently been reported that the survival times on dry surfaces of *A. baumannii* biofilm-forming strains are longer than those of non-biofilm-forming ones [38]. However, the collection analysed in this study did not reveal a strong correlation between biofilm formation and survival to desiccation, except for strains assigned to ST78 and two out of three strains assigned to ST25. A possible explanation for this discrepancy is that this correlation depends on the strains and/or culture conditions used.

The data obtained in this study also indicate that *A. baumannii* clonal lineages differ in the ability to adhere to alveolar epithelial cells. In particular, adherence to A549 cells was higher for strains assigned to ST2, ST25 and ST78, and a positive correlation exists between adherence to epithelial cells and biofilm formation in these strains. This is consistent with a previous report showing a positive association between biofilm production and adhesiveness to epithelial cells in MDR clinical isolates of *A. baumannii*[20]. Therefore, it can be speculated that common or partly overlapping cellular mechanisms might control biofilm growth on abiotic surfaces and adherence to epithelial cells. In partial support of this hypothesis, it was recently shown that the biofilm-associated protein Bap plays a role in both biofilm formation and adherence to human epithelial cells [21]. Our study also demonstrated that *A. baumannii* strains assigned to ST78 have a higher ability to invade A549 human bronchial epithelial cells than other genotypes. However, since the three strains assigned to ST78 belong to the same major PFGE type, it cannot be excluded that differences in the capacity to invade epithelial cells are strain-specific rather than lineage-specific.

No significant differences in the killing of *G. mellonella* larvae, measured as the LD_50_ at the 72 h post-infection time point, were found among strains assigned to the distinct genotypes, with the exception of strains ATCC 19606^T^ (ST52) and 3868 (ST15), which showed significantly lower virulence. However, strain-specific differences were observed in the rate of killing, expressed as the ratio between LD_50_ values determined at 24 and 72 h. Killing was delayed for strains AYE (ST1) and 3990 (ST2) and, to a lesser extent, 3865, 4190 (both ST25) and 3911 (ST78) (LD_50_ ratio 5.3-12.2; Table 2 and Additional file 2: Figure S1). Overall, the LD_50_ values determined for our collection of isolates is in the same order of magnitude as that previously reported for prototypic *A. baumannii* strains of clinical origin (AYE, ACICU and ATCC 17978) [23].

The data just discussed indicate that, in spite of some interclonal variation, biofilm formation, adherence to epithelial cells and resistance to desiccation are virulence traits shared by *A. baumannii* clinical strains, irrespective of their belonging to the major or minor lineages. This is consistent with previous work [10,18,23,38], including some recent publications showing that several genes associated with *A. baumannii* pathogenicity are more conserved in the *A. calcoaceticus-baumannii* (Acb) complex, and especially in *A. baumannii*, than in other *Acinetobacter* genomes, and that the most successful *A. baumannii* strains, although epidemiologically and genotypically different, share a common set of virulence-related traits in their core genome [25-27].

## Conclusions

In conclusion, although epidemic *A. baumannii* clonal lineages show common virulence traits, they differ in the expression of virulence-associated phenotypes. In particular, *A. baumannii* strains belonging to IC-I showed higher resistance to desiccation, strains belonging to IC-II showed higher adherence to both biotic and abiotic surfaces, strains belonging to emerging genotypes ST25 and ST78 showed elevated resistance to desiccation, adherence to biotic and abiotic surfaces. Also, to the best of our knowledge, this is the first study to identify common *A. baumannii* virulence-associated phenotypes (resistance to desiccation, adherence to biotic and abiotic surfaces and alveolar epithelial cell invasion) in epidemic genotypes other than the three main ICs. Non-IC genotypes have increasingly been reported as responsible of hospital epidemics [5], and our findings are a first step towards the elucidation of virulence strategies that might have favoured the spread and persistence in the hospital environment of these emerging sequence types.

## Abbreviations

MLST: Multilocus sequence typing; ST: Sequence type; IC: International clonal lineages; MDR: Multi-drug-resistant; XDR: Extensively-drug-resistant; PFGE: Pulsed-field gel electrophoresis; LB: Luria-Bertani broth; M9: Mueller Hinton broth; OD: Optical density; PBS: Phosphate-buffer saline solution; CFU: Colony-forming units; LD50: Lethal dose 50%; r: Pearson’s correlation coefficient.

## Competing interests

The authors declare that they have no competing interests.

## Authors’ contributions

Conceived and designed the experiments: MG, LA, MT, PV, RZ. Performed the experiments: MG, LA, VM. Analyzed the data: MG, LA, PV, RZ. Wrote the paper: MG, LA, PV, RZ. All authors read and approved the final manuscript.

## Pre-publication history

The pre-publication history for this paper can be accessed here:

http://www.biomedcentral.com/1471-2334/13/282/prepub

## Supplementary Material

Additional file 1: Table S1 Resistance to desiccation of *A. baumannii* strains. Values are means ± standard deviations of three independent experiments done in duplicate. Click here for file

Additional file 2: Figure S1 Kaplan-Meier survival plots of *G. mellonella* larvae infected with the different *A. baumannii* strains. Click here for file
