# Supplementary figures and images for: Virulence-related traits of epidemic Acinetobacter baumannii strains belonging to the international clonal lineages I-III and to the emerging genotypes ST25 and ST78
Source: BMC Infect Dis. 2013 Jun 20;13:282. doi: 10.1186/1471-2334-13-282 (PMC3691691; doi:10.1186/1471-2334-13-282)

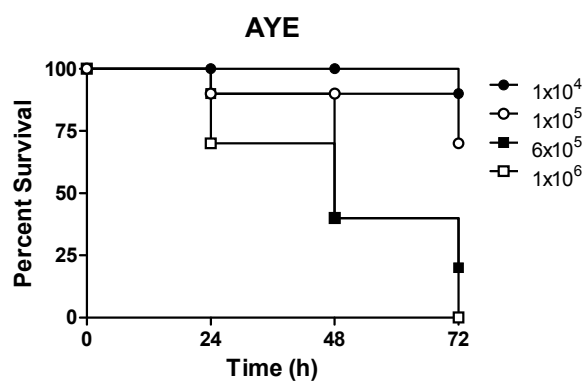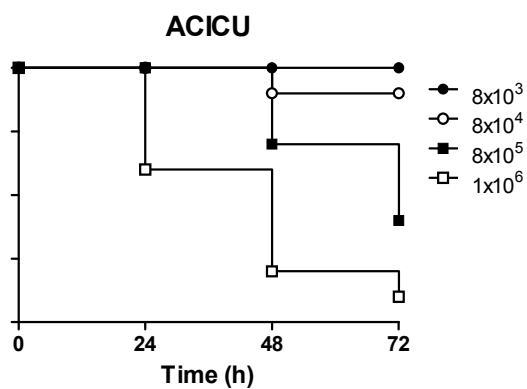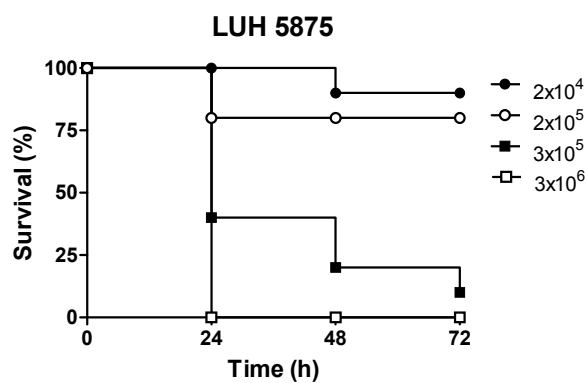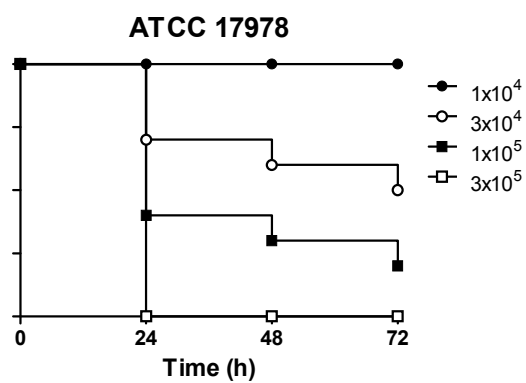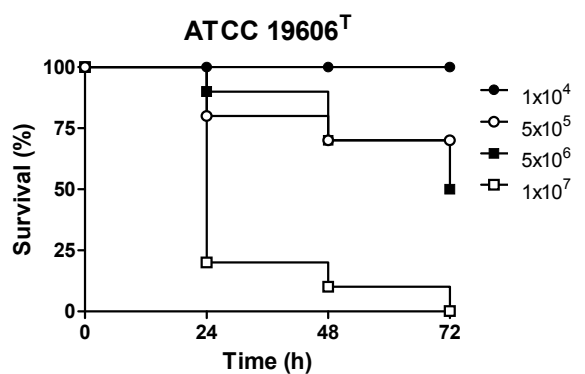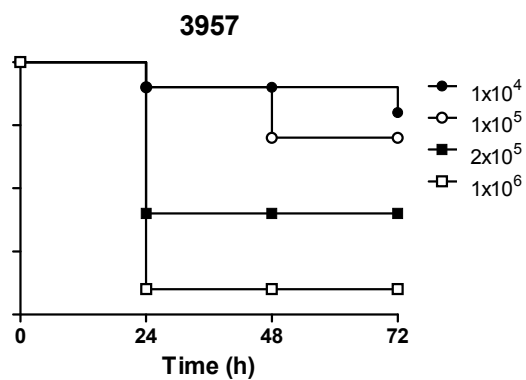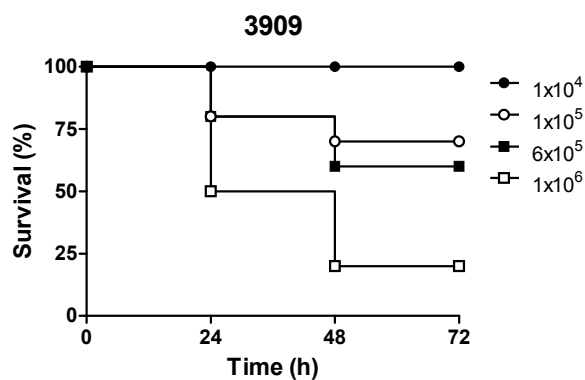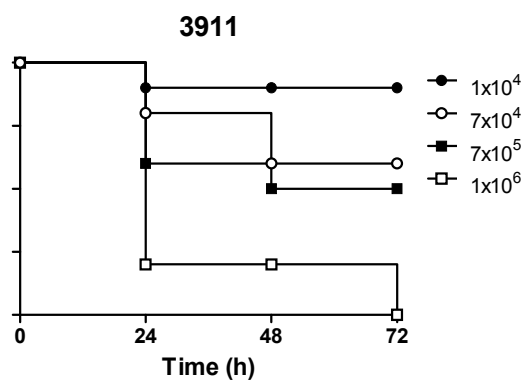

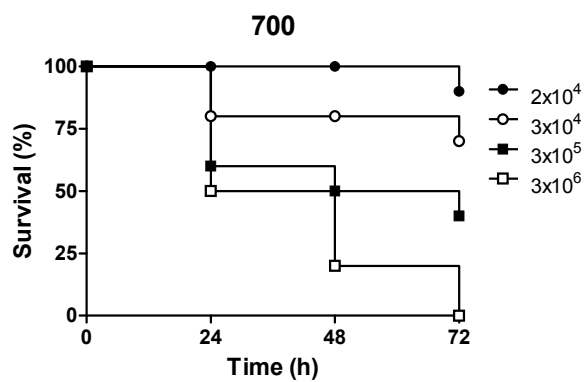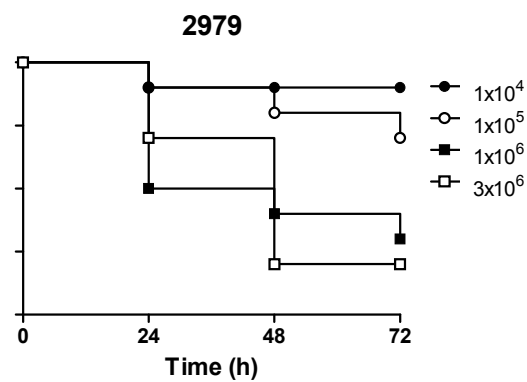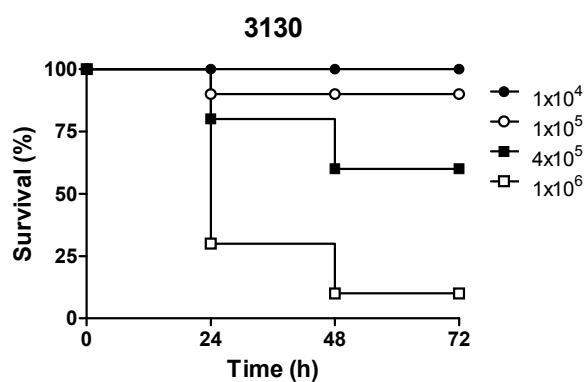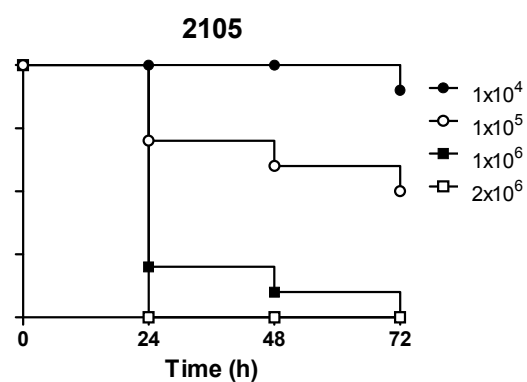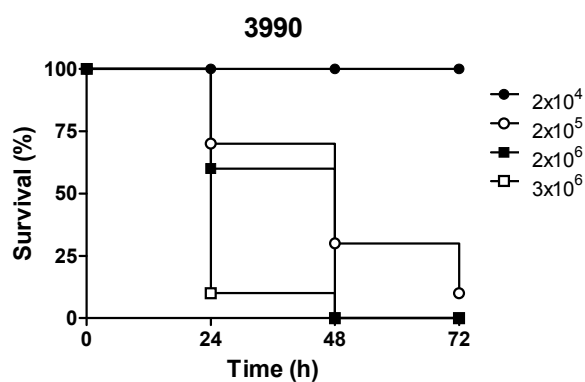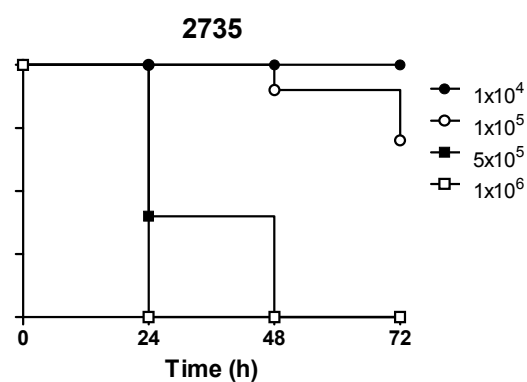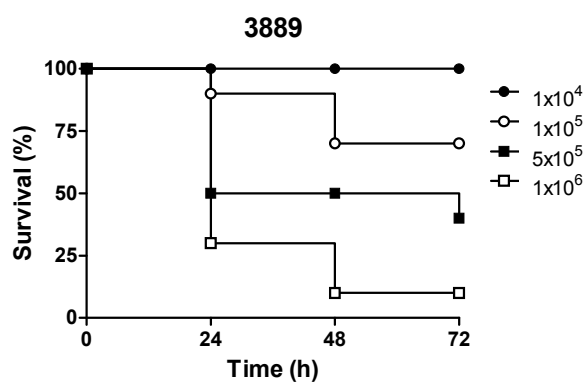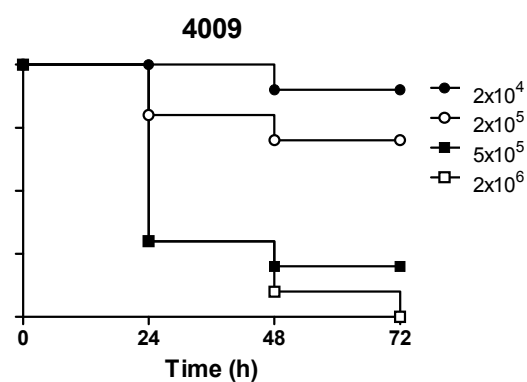

4025

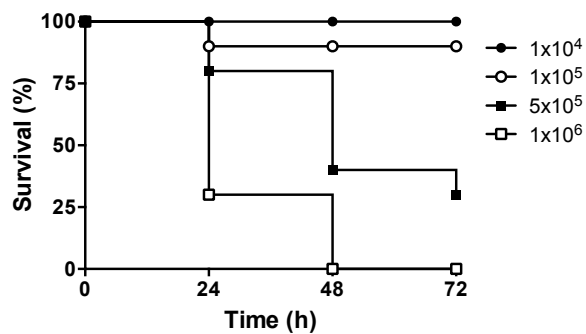

4026

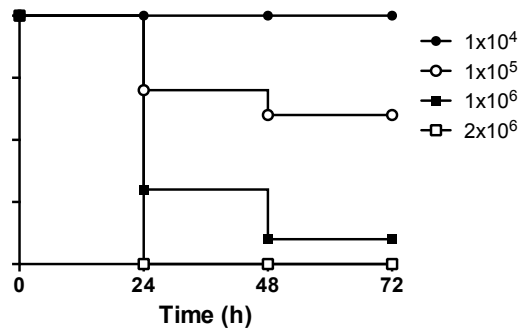

3890

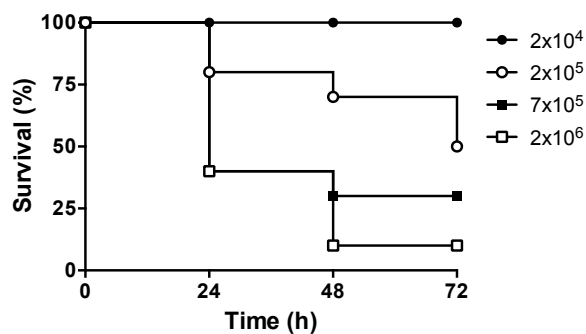

4190

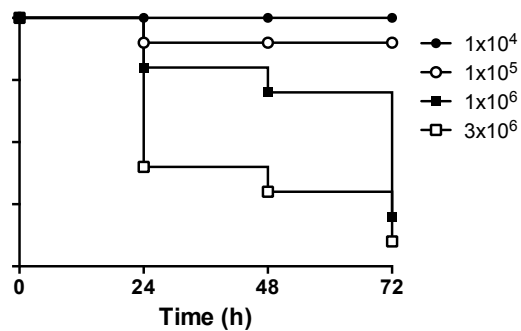

3865

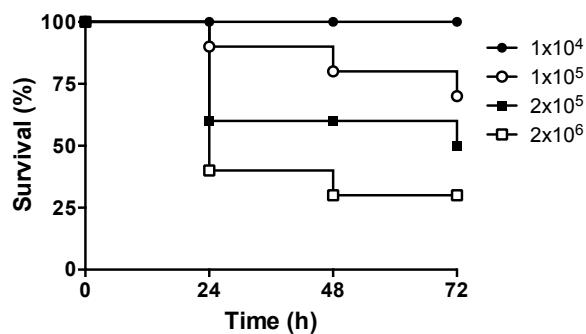

3871

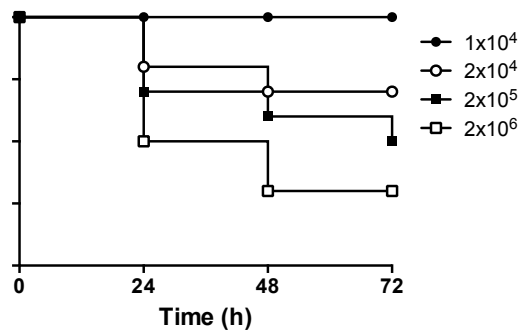

3868

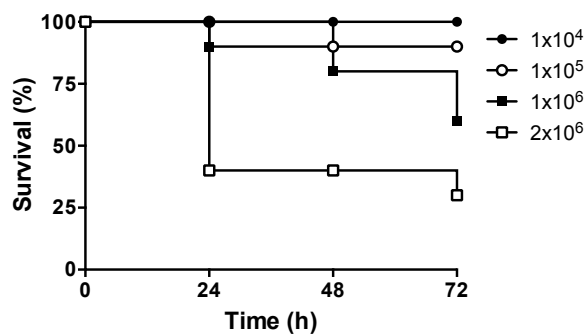

Supplement: Additional file 2: Figure S1 — Kaplan-Meier survival plots of G. mellonella larvae infected with the different A. baumannii strains. [file 1471-2334-13-282-S2.pdf]
